# Durability Assessment of Alkali-Activated Geopolymers Matrices for Organic Liquid Waste Immobilization

**DOI:** 10.3390/ma18133181

**Published:** 2025-07-04

**Authors:** Rosa Lo Frano, Salvatore Angelo Cancemi, Eleonora Stefanelli, Viktor Dolin

**Affiliations:** 1 Dipartimento di Ingegneria Civile e Industriale, Università di Pisa, Largo Lucio Lazzarino 2, 56122 Pisa, Italy; salvatore.cancemi@unipi.it (S.A.C.); eleonora.stefanelli@unipi.it (E.S.); 2 State Institution “Institute of Environmental Geochemistry of National Academy of Sciences of Ukraine”, 34a, Palladin Av., 03680 Kyiv, Ukraine; vdolin@ukr.net

**Keywords:** geopolymer, liquid organic waste, thermal ageing, fire resistance, radioactive waste management

## Abstract

This study investigates the mechanical and microstructural performance of three alkali-activated geopolymer formulations, constituted of metakaolin (MK), blast furnace slag (BFS), and a ternary blend of MK, BFS, and fly ash (MIX), for the immobilization of simulated radioactive liquid organic waste (RLOW). Thermal ageing tests were performed to evaluate geopolymer durability, including fire exposure (800 °C) and climatic chamber cycles (from −20 to 40 °C). Characterization through thermogravimetric analysis (TGA), compression tests, and scanning electron microscopy with energy-dispersive spectroscopy (SEM-EDS) was carried out to assess material degradation after thermal ageing. Preliminary results showed substantial strength and microstructural degradation in oil-loaded specimens after cyclic climatic ageing, while fire-exposed blank matrices retained partial mechanical integrity. BFS matrices exhibited the best thermal resistance, attributable to the formation of Ca-Al-Si-hydrate (C-A-S-H) gels. These findings support the use of optimized geopolymer formulations for safe RLOW immobilization, while contributing to the advancement of knowledge on sustainable and regulatory-compliant direct conditioning technology.

## 1. Introduction

Radioactive waste management requires that the waste be safely immobilized in stable and durable matrices, whatever predisposal or disposal conditions. In pursuing this objective, it is therefore essential to identify, classify, and characterize the nuclear wastes and/or materials from industrial, research, and medical applications.

Identifying the best waste route is crucial for pre-disposal and requires the availability of temporary storage or disposal facilities. Despite significant progress in radioactive waste management worldwide, effective solutions for the full spectrum of radioactive waste require further investigation. This was the main objective of the EU PRE-DISposal management of radioactive waste (PREDIS) [1] project, which focused on innovation and innovative technologies for the safer, more efficient, economical, and environmentally friendly management of radioactive waste ILW/LLW. Particularly in its WP5, options for direct conditioning of radioactive liquid organic wastes (RLOW) by employing innovative geopolymers and related alkali-activated materials have been developed and investigated. As is widely known, such wastes include a variety of organic extraction solvents (large volumes), various (e.g., lubricant, hydraulic fluid, etc.) contaminated oils, liquid scintillation cocktails, and miscellaneous solvents (e.g., tributyl phosphate (TBP), or TBP plus a diluent such as dodecane) (small volumes) [2,3,4,5,6], etc., as given in Table 1 below.

RLOWs are highly mobile which contributes to spreading contamination; thus, they must be effectively immobilized. Moreover, many of them are volatile and combustible or support the combustion of other wastes. Many organic fluids are immiscible with water (“non-aqueous phase liquids”) and could rapidly migrate into the environment (the lighter fraction can float on water, whereas the dense fraction cannot). Due to the complex and variable physical-chemical properties, many countries have considered different techniques for conditioning RLOW. Since to date, an adequate method for treatment has not yet been defined, in many countries, such waste is stored awaiting conditioning.

The safe management and conditioning of RLOWs are challenging from a regulatory requirements point of view and the waste-form durability (integrity).

In the past, traditional conditioning approaches using Ordinary Portland Cement (OPC) have been found to inadequately accommodate the chemical complexity of RLOW, often resulting in poor interactions between the organic components and the cementitious matrix, delayed setting times, and diminished mechanical properties [6,7]. Consequently, alternative conditioning matrices based on geopolymers and alkali-activated materials (AAMs) have gained attention due to their superior durability, enhanced chemical stability, and the ability to incorporate high loading levels of organic wastes without significant adverse effects on their structural integrity [8,9].

Geopolymers are typically synthesized by the alkaline activation of aluminosilicate sources, such as metakaolin (MK), or industrial wastes, such as fly ash (FA) and blast furnace slag (BFS), resulting in a three-dimensional network structure that exhibits excellent mechanical performance, low permeability to liquids, high fire resistance, and chemical inertness [8,10].

In the context of RLOW conditioning, the direct incorporation of waste oils into geopolymeric formulations offers several advantages over traditional encapsulation methods. This approach minimizes pretreatment requirements such as pre-emulsification or pre-impregnation, thereby reducing processing complexity and cost [11,12]. Moreover, the direct conditioning of RLOW within geopolymer matrices not only provides a robust barrier against radionuclide migration but also leverages the inherent fire resistance and thermal stability of geopolymers to mitigate the risks associated with organic compounds under high-temperature conditions [13].

Over the last three decades, alkali-activated aluminosilicate binders (geopolymers) have emerged as a promising alternative to ordinary Portland cement for conditioning challenging waste streams. The author [14] demonstrated their ability to chemically trap hazardous species and form dense, low-permeability matrices. Subsequent studies confirmed efficient immobilization of a broad spectrum of radionuclides, including Cs^+^, Sr^2+,^ and actinides, while retaining excellent chemical stability [15,16]. More recently, several authors have focused on liquid organic waste: Zhang et al. evaluated direct incorporation of scintillation cocktails in metakaolin-based pastes [17]. A comprehensive overview of these efforts is given in the PREDIS Deliverables 5.2, 5.3, 5.5, and 5.6 [5,18,19,20], which outline the remaining knowledge gaps in long-term performance under accidental thermal loads. Several consortium partners in the PREDIS Project carried out systematic leaching campaigns on geopolymer and alkali-activated matrices loaded with representative RLOW streams. These tests employed ^63^Ni, ^14^C. The leaching behavior was also studied on specimens irradiated at 200 kGy, to investigate possible degradation due to absorbed dose [18]. Despite this progress, systematic data on the combined effects of thermal cycling and short-duration fire exposure on geopolymers are still lacking. Investigations of the effects of waste loading on microstructure, rheology, and mechanical behavior have demonstrated that appropriately tailored geopolymer matrices can achieve desirable properties even at high waste contents, whether formulation parameters—such as the Si/Al ratio, type of alkaline activator, and use of suitable additives—are rigorously controlled [3,13]. In particular, detailed microstructural examinations using scanning electron microscopy (SEM) have revealed that the incorporation of RLOW can be achieved without compromising the homogeneity of the matrix, as long as proper emulsification and mixing protocols are followed. Thermal analyses via differential scanning calorimetry (DSC) and thermogravimetric analysis (TGA) further confirm that the geopolymerization process remains robust in the presence of organic additives, retaining a high degree of thermal stability and predictable decomposition behavior under fire exposure [10].

The objective of this study is to characterize the performance of new geopolymer-based and AAM conditioning matrices specifically designed for the direct incorporation of RLOW over long timeframes by performing TGA, SEM analysis, compressive strength test, and fire/elevated temperature test. These tests focused primarily on analyzing the durability of such matrices, as well as the ability to withstand accidental and/or seasonal thermo-mechanical stress. It is worth noting that to allow conducting experiments, without worrying about radiation protection concerns, non-radioactive representative samples or RLOW surrogates were used.

The study provides the interrelationship between waste loading, microstructural integrity, thermal behavior, and mechanical performance, thereby establishing a scientific basis for the implementation of RLOW direct conditioning solutions in nuclear waste management. Ultimately, insights gained herein are expected to advance the development of sustainable, cost-effective, and regulatory-compliant waste treatment technologies, further solidifying the role of geopolymers and alkali-activated materials in the nuclear industry.

## 2. Materials and Methods

### 2.1. Geopolymer Preparation

The three (geopolymer) matrix type formulations investigated in this paper, developed by the direct conditioning route, were: metakaolin-based (MK), blast furnace slag-based (BFS), and a ternary mixed system (MIX) composed of fly ash (FA), BFS, and MK.

All the samples were synthesized by different project partners, and the formulation compositions were selected and optimized according to preliminary incorporation tests conducted in a previous study [2].

Table 2 summarizes the composition and preparation details for each geopolymer formulation, including both blank and oil-loaded samples. The chemical composition of the raw materials employed for the three geopolymers formulation is reported in Table S1 of the Supplementary Materials File.

The MK formulation was developed by the UK’s National Nuclear Laboratory (NNL) employing high-purity metakaolin (Metamax^®^, BASF, Cheshire, UK) as the aluminosilicate precursor, composed of 54.1 wt% of SiO_2_ and 42.5 wt% of Al_2_O_3_. The activating solution was prepared by dissolving potassium hydroxide pellets (KOH, ≥85%, Scharlab, Vimodrone, Italy) in deionized water, followed by the addition of potassium silicate solution (Betol^®^ K5020T, Woellner, Germany; composition: 30.0 wt% SiO_2_, 18.5 wt% K_2_O, and 51.5 wt% H_2_O). The molar ratios of the activating system were adjusted to SiO_2_/K_2_O = 1.2 and H_2_O/K_2_O = 13. Two different surrogate RLOWs were added to the MK-based matrix: Nevastane EP 100 (TotalEnergies Lubricants, Singapore) lubricating oil (density 0.871 g/cm^3^) and tributyl phosphate (TBP)/dodecane (30/70 vol/vol) solvent (density 0.850 g/cm^3^). The overall composition of MK-based geopolymers with and without oil addition is reported in Table 2.

The BFS matrix was prepared by the Belgium Studiecentrum voor Kernenergie/Centre d’Etude de l’Energie Nucléaire (SCK CEN) using finely ground granulated blast furnace slag (Ecocem Benelux, Beveren, Belgium; composition: 33.6 wt% SiO_2_, 8.7 wt% Al_2_O_3_, and 49.0 wt% CaO) and quartz sand as filler. The activator comprised sodium hydroxide solution (NaOH 10 M), sodium silicate (Na_2_SiO_3_), and demineralized water.

The RLOW simulant used was TBP/dodecane solvent. Tween^®^ 80 (0.5 vol% relative to oil) was employed as a surfactant to promote dispersion and emulsion stability. Oil-loaded samples were prepared with a final oil content of 20 vol% (Table 2).

The MIX system was produced by the National Science Centre Kharkov Institute of Physics and Technology (KIPT) in Ukraine, incorporating a blend of fly ash (34 wt%), BFS (20 wt%), and MK (14 wt%) as precursors (Table 2). The chemical composition of Ukrainian raw materials employed for the MIX formulation is exhaustively detailed in [2]. The alkaline activator consisted of potassium silicate solution (Betol^®^ K5020T, 11 wt%), KOH (9 wt%), and water (12 wt%). Castament FW-10 (solid polyethylene glycol-based additive, BASF) was employed as a plasticizer and surfactant. The surrogate RLOW added was Shell Spirax (Shell plc, London, UK) lubricating oil (density 0.882 g/cm^3^).

**Figure 1:**
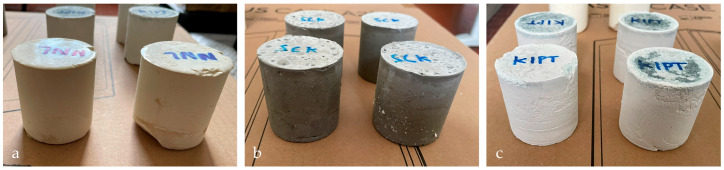
Images of samples of geopolymer formulations with RLOW simulants: (**a**) MK-based, (**b**) BFS-based, and (**c**) MIX-based.

All the geopolymer formulations were mixed using a planetary mixer under controlled conditions (refer to [2]) and cast into cylindrical molds of 50 mm in diameter and 50 mm in height (Figure 1). Specimens were sealed to prevent evaporation and cured for 28 days at 20 °C under > 90% relative humidity in a climatic chamber before characterization. It was explored a wide waste-loading window was explored under the project [1]. Robustness trials demonstrated that direct encapsulation of representative lubricating oils could be achieved up to 40 vol %—and in isolated cases even 50 vol %. However, the 20 vol% was chosen as the practical upper limit for all formulations.

### 2.2. Thermal Ageing Protocols

Accelerated climatic chamber ageing tests were performed to simulate environmental temperature fluctuations on MK-based and MIX-based specimens (blank and oil-loaded). Samples were subjected to 15 thermal cycles according to CEI EN 60068-2-14 standard [21] (Nb test), simulating thermal fatigue between −20 and 40 °C for a total aging time of 100 h. Each cycle involved heating from −20 to 40 °C at 3 °C/min, holding at 40 °C for 3 h, cooling from 40 to −20 °C at 3 °C/min, and holding at −20 °C for 3 h.

High temperature resistance was evaluated by carrying out fire tests according to [20] by heating the MK-based, the BFS-based, and the MIX formulation samples without oil at 800 °C. Thermocouples type K are allowed to monitor test conditions, check the correct execution of the test, and acquire the data. The presence of RLOW simulants with low flash points (146–240 °C) impeded fire tests execution on oil-loaded samples due to safety reasons. Samples were therefore held at peak temperature for 30 min and left to cool naturally until ambient conditions were reached [22].

### 2.3. Characterization Techniques

Thermal gravimetric analysis (TGA) was performed on samples prior to the cyclic thermal ageing to verify the degradation behavior using a Pyris 1 analyzer (Perkin Elmer, Waltham, MA, USA). About 15 mg of powdered samples were tested under air flow using the following thermal program: heating from 30 to 60 °C at 20 °C/min, maintaining 60 °C for 30 min, and then heating from 60 to 800 °C at 20 °C/min. The weight loss profiles were recorded over time to identify dehydration and organic decomposition steps. Differential Scanning Calorimetry (DSC) analysis was carried out prior to and after cyclic thermal ageing on powdered MK-based and MIX-based specimens to determine the specific heat capacity (Cp) of the samples in accordance with the EN ISO 11357-1 standard [23]. The thermal program consisted of three consecutive stages performed under nitrogen atmosphere: (i) first heating from −20 to 120 °C at 20 °C/min, (ii) cooling from 120 to −20 °C at −20 °C/min, and (iii) second heating from −20 to 120 °C at 20 °C/min. Cp values were extracted from the second heating cycle to eliminate the influence of thermal history and ensure reproducibility of the results.

Compressive strength measurements were carried out at 20 °C on the fresh, thermal-aged, and post-fired cylindrical samples using an MTS Landmark^®^ 370.25 (MTS Systems S.r.l., Torino, Italy) testing machine equipped with a 250 kN load cell. A crosshead speed of 5 mm/min was applied. All the compressive measurements were conducted on a minimum of 3 specimens for each sample, and the average values were reported.

The morphological analysis was conducted on all samples reference without RLOW by scanning electron microscopy (SEM) using a FEI Quanta FEG 450 (FEI Inc., Hillsboro, OR, USA) to examine microstructural morphology pre- and post-fire exposure, and after cyclic thermal ageing. Small fragments from each sample group were platinum-coated and analyzed under high vacuum conditions. Blank specimens of MK, BFS, and MIX matrices were analyzed to assess the effects of thermal ageing and fire-induced damage.

## 3. Results and Discussion

### 3.1. Characterization of Geopolymer Samples

Before applying ageing protocols, geopolymer formulations were characterized by TGA, SEM, and by performing compressive strength tests to establish a baseline for comparison and to assess the microstructural and thermal behavior of the unaged materials.

**Figure 2:**
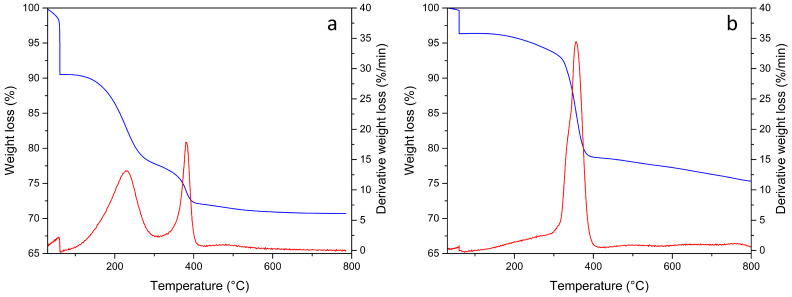
TGA analysis of samples (**a**) MK-T and (**b**) MIX-S: TG (blue lines) and DTG (red lines) profiles in air.

MK-T and MIX-S specimens with oil addition were subjected to TGA experiments to verify the thermal degradation behavior, and the results are reported in Figure 2 in terms of both thermogravimetric (TG) and derivative (DTG) curves. During the isothermal step at 60 °C, the MK-T sample shows a mass loss of 9.4% related to moisture, while the MIX-S exhibited a lower moisture content of 3.6%, indicating a reduced hygroscopic behavior. During the thermal treatment from 60 to 800 °C, both samples exhibit a total weight loss of about 20%, which is attributable to the combustion of the organic oils incorporated in the geopolymer matrices. MIX-S sample results are more stable at higher temperatures as the weight loss before 300 °C is only 8.6%, and this is attributed to the RLOW surrogate used for geopolymer preparation that presents a unique degradation at a peak temperature around 357 °C. Whereas, the MK-T sample presents a minor high-temperature resistance due to the combustion of dodecane, which occurs in the range 120–300 °C. The second peak in the DTG curve at about 380 °C could be ascribed to the combustion of TBP.

**Figure 3:**
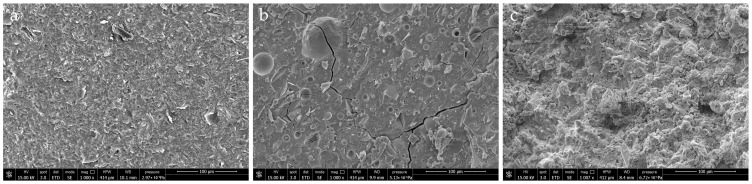
SEM images of the tested geopolymer matrices without RLOW simulants: (**a**) MK, (**b**) BFS, and (**c**) MIX.

SEM analysis was conducted on no oil-loaded geopolymer matrices due to safety constraints, and the obtained images are reported in Figure 3. The blank MK sample (Figure 3a) shows a relatively homogeneous and dense microstructure, typical of metakaolin-activated geopolymers using potassium-based activators [13]. In contrast, BFS formulation reveals a heterogeneous microstructure characterized by the presence of numerous spherical to hemispherical voids dispersed within the matrix (Figure 3b). This structure is morphologically consistent with entrapped gas, a commonly reported effect in alkali-activated materials and geopolymers, particularly when slag is used as the sole precursor [24]. Such porosity is often attributed to hydrogen gas evolution during the dissolution of metallic aluminum traces present in blast furnace slag under highly alkaline conditions, as well as from the entrapment of water vapor or dissolved gases during the geopolymerization process [25]. The observed pores vary in size and distribution, suggesting non-uniform degassing and/or incomplete consolidation during the setting process. Despite this porosity, the surrounding matrix appears relatively dense, indicating that the geopolymerization reaction progressed substantially, possibly forming calcium-aluminosilicate hydrate (C-A-S-H) phases typical of slag-based systems [26]. SEM image of MIX sample (Figure 3c) shows a heterogeneous microstructure with unreacted fly ash particles embedded in a dense matrix, consistent with systems where multiple precursors contribute distinct reactivity and gel phases. BFS provides early calcium-rich reaction products that promote rapid setting and strength development, while MK and FA contribute to a finer pore structure and increased chemical stability over time [25,26].

Compression tests were carried out to evaluate the strength of samples before thermal ageing. The results of the compression tests conducted on both blank and oil-loaded samples prior to thermal ageing are reported in Table 3.

Figure S1 in the Supplementary File shows the execution of the compression tests. The brittle rupture mode with projection of fragments is visible. The mean maximum value of the compression stress obtained for samples without oil ranges is between 11 and 13 MPa, with a high standard deviation among the three samples of MK. Oil-loaded samples exhibit a lower ultimate strength with respect to the blank matrices, especially for MIX-S samples that achieve only 5.63 MPa. These results suggest that the oil addition to the MIX formulation could compromise the matrix integrity, probably due to an increased porosity or hindered gel development during curing. Among MK-based samples, MK-N and MK-T showed a slight compressive strength reduction (10.72 and 9.13 MPa, respectively), indicating that moderate thermal exposure does not significantly impair the strength. However, the MK-N sample had the lowest strength in the MK series (9.13 MPa), suggesting altered reaction kinetics or incomplete gelation, possibly due to the oil addition. Table 3 evidences that the direct encapsulation of 20 vol % simulated oil (RLOW) produces a composition-dependent mechanical penalty: the metakaolin reference loses only 6–20% of its strength when oil is added, whereas the ternary MIX formulation undergoes a drastic about 53% reduction, confirming that oil inclusion amplifies porosity and interfere with gel consolidation most severely in chemically heterogeneous binders.

### 3.2. Climatic Chamber Ageing Tests

The post-thermal ageing performance of the geopolymer matrices was analyzed to determine their mechanical strength, thermal stability, and microstructural integrity after exposure to accelerated ageing environments. Accelerated ageing tests simulate long-term environmental temperature fluctuations that waste containment systems may experience and help assess the material’s durability over time.

Figure S2 in the Supplementary File shows the execution of thermal ageing tests in a climatic chamber, exposing the samples to 15 cycles between −20 and 40 °C. After thermal ageing, compression tests were performed on MK-based and MIX-based specimens with and without oil, and the results in terms of crush load and ultimate stress are reported in Table 4. As shown, thermal cycling caused significant degradation in MK samples, both blank and oil-loaded, whereas MIX-based geopolymers showed a slight increase in the ultimate stress as compared to the non-thermal treated samples. The slight increase in compressive strength observed post-ageing may be associated with continued geopolymerization or secondary gel formation during the thermal cycles [27,28].

MK and MIX samples without oil prior to and after the thermal ageing tests were analyzed by SEM-EDS to assess potential changes in surface composition, elemental migration, or degradation of the geopolymer network, thereby providing insights into the chemical stability and integrity of the matrices before and after exposure to ageing protocols.

**Figure 4:**
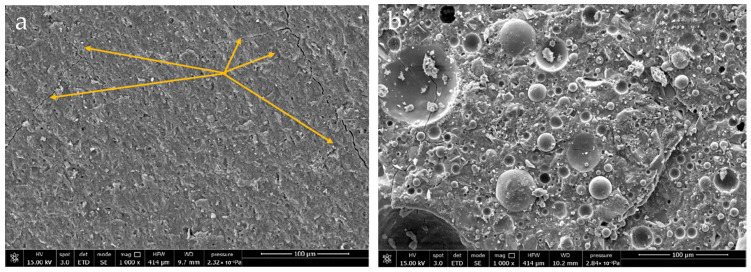
SEM images of the geopolymer matrices without RLOW simulants after cyclic thermal ageing in climatic chamber: (**a**) MK, and (**b**) MIX [18].

SEM images of MK and MIX samples without oil aged by thermal cycling (Figure 4) supported the compression test results. Figure 4a displays the presence of micro-cracks in the MK aged sample, clearly visible at the location indicated with yellow arrows, which could explain the drastic reduction in compressive strength. Whereas the aged MIX sample (Figure 4b) presents an almost unchanged microstructure.

**Figure 5:**
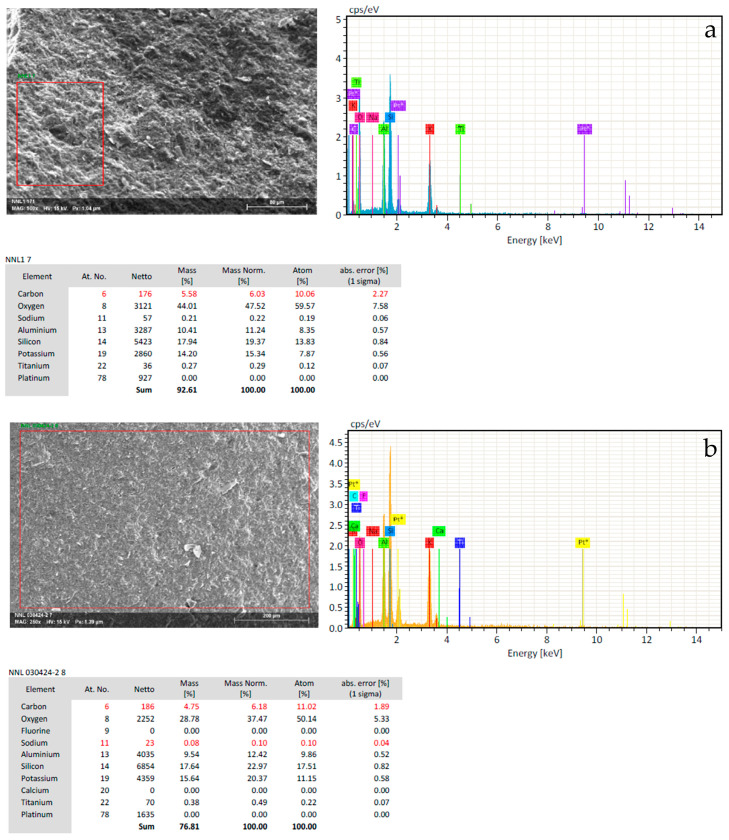
SEM-EDS analysis of MK samples without RLOW simulants: (**a**) before and (**b**) after cyclic thermal ageing in climatic chamber.

For MK-based specimens subjected to climatic ageing, the elemental composition was detected by Energy Dispersive Spectroscopy (EDS) analysis (Figure 5). In the unaged sample, the EDS analysis confirms the presence of the expected geopolymer network, with elevated oxygen content (44.01 wt%), and significant concentrations of silicon (17.94 wt%) and aluminum (10.41 wt%).

A substantial potassium content (14.20 wt%) was also detected, consistent with the use of potassium silicate and KOH in the activating solution. After the cyclic thermal ageing, a slight decrease in silicon and aluminum concentration could be observed, along with a notable reduction of the oxygen content to 28.78 wt%. This decrease may indicate a partial dehydration of the gel with a reorganization of the amorphous network into a more thermodynamically stable [19,20]

Despite these shifts, the maintenance of the Si/Al ratio suggests that the core aluminosilicate framework remains chemically stable after thermal cycling, although physical degradation was observed (Figure 4a) and through mechanical testing (Table 4).

**Figure 6:**
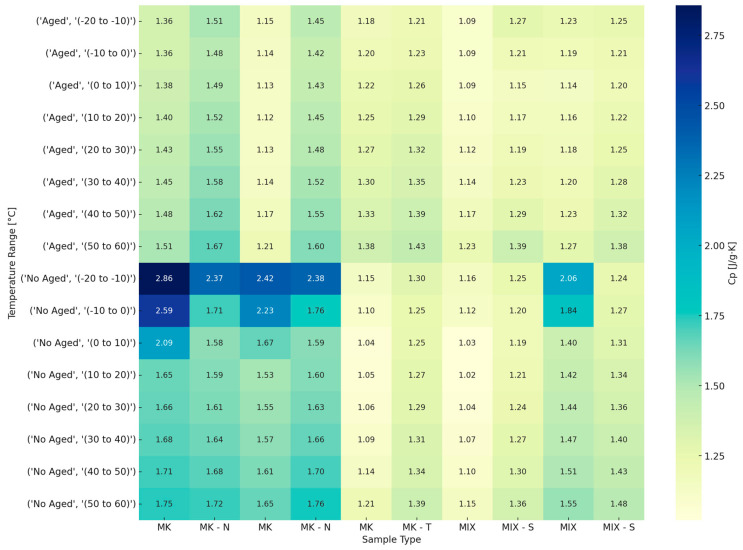
Heatmap of specific heat capacity for Aged and No-Aged formulations.

Figure 6 presents a comprehensive heatmap illustrating the specific heat capacity (Cp) of MK and MIX samples in the range of −20 °C to 60 °C. The samples are categorized by both formulation type and ageing condition (Aged vs. Non-Aged). This heatmap enables a comparative analysis of thermal storage capacity change under different chemical compositions and environmental exposures.

A systematic reduction in Cp is observed in all aged samples when compared to those not aged. This reduction is marked in MK and MK-N formulations, which show declines exceeding 1.0 J/g·K at lower temperatures (e.g., −20 to −10 °C). The observed behavior is consistent with ageing-induced phenomena:

- Structural densification and phase stabilization, reducing degrees of freedom for heat storage.
- Evaporation or decomposition of volatile organic components.
- Reduction in pore-bound water content, particularly relevant for alkali-activated matrices.

MIX-based samples, particularly MIX-S, which contain ShellSpirax, generally maintain higher and more stable Cp values across the entire temperature range, even after ageing. This suggests that the presence of BFS and FA supports a thermally resilient structure with improved moisture retention. By contrast, MK-T formulations show consistently lower Cp values, suggesting potential incompatibility of TBP with the MK matrix or volatilization during ageing.

MK-N shows intermediate values of Cp, indicating thermal benefit due to organic inclusion, but not as effectively retained under ageing as seen with MIX-S. Across nearly all formulations, Cp increases with temperature, which is consistent with the general behavior of amorphous materials. However, the rate of increase is lower in the aged samples, particularly in MK and MK-T, where the thermal response appears to saturate around 1.5 J/g·K, even at elevated temperatures.

Notably, fresh MK formulations display anomalously high Cp values at low temperatures (up to ~2.8 J/g·K), potentially symptomatic of entrained moisture or unreacted organics. This suggests a need for further thermal characterization to study hydration state or organic degradation dynamics.

### 3.3. Fire Resistance

**Figure 7:**
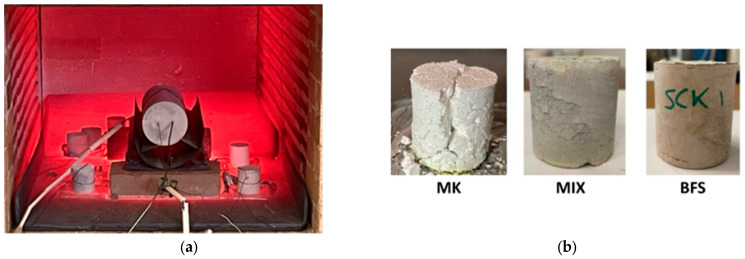
Fire test execution (**a**) and sample shape at the end of the fire test (**b**) [18].

Figure 7a shows the fire test execution. Visual inspection after post-fire tests (Figure 7b) revealed surface cracking (hourglass shape) and spalling in MK samples, while BFS and MIX blank formulations have maintained cohesion with only minor delamination. The MK sample exhibited visible surface cracking and volumetric instability, which are indicative of shrinkage-induced distress.

Crack development path, almost vertical, even not perfectly straight, extends from the upper surface downwards, suggesting tensile failure is occurring. The resulting damage is so representative of the microstructural brittleness of the tested samples. Cracking was due to drying shrinkage stress exceeding the tensile stress of the material. During test execution, tensile stress appears mainly along the sample’s diameter, where the tangential stress exceeds the uniaxial tensile strength, leading to failure.

Therefore, shrinkage not only compromises the dimensional stability of the material but also diminishes its load-bearing capacity and resistance to spalling under thermal stress. The shrinkage effects are certainly worsened by the high porosity of the material.

The compressive stress of samples after fire-like (AFL) conditions is summarized in Table 5. BFS-based samples demonstrate moderate strength, retaining 30.05 and 19.53% of their original strength, with residual compressive values of 3.15 and 2.00 MPa, respectively. Although strength losses of 70–80% were recorded, the BFS matrix did not undergo complete disintegration, suggesting a more thermally stable C-A-S-H network that partially resists high-temperature exposure.

The MIX formulation displays superior fire resistance among the three samples, preserving 58.81 and 32.13% of the initial compressive strength, with post-fire values of 6.02 and 4.71 MPa, respectively. These results suggest that the hybrid matrix, combining MK, BFS, and fly ash, benefits from synergistic effects that enhance thermal stability and mechanical integrity under fire exposure.

The findings confirm the critical role of precursor composition in influencing the fire resistance of geopolymer waste forms. In addition, the fire test highlights the need for enhanced thermal stabilization strategies, including the incorporation of thermally stable aggregates, reinforcement fibers, or heat-resistant additives to improve the high-temperature performance of geopolymer composites.

**Figure 8:**
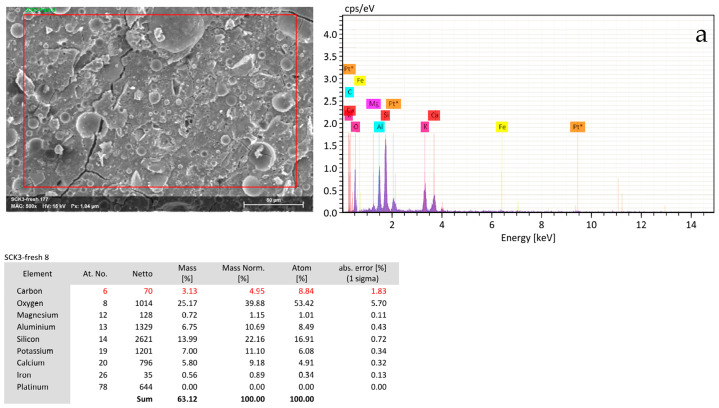

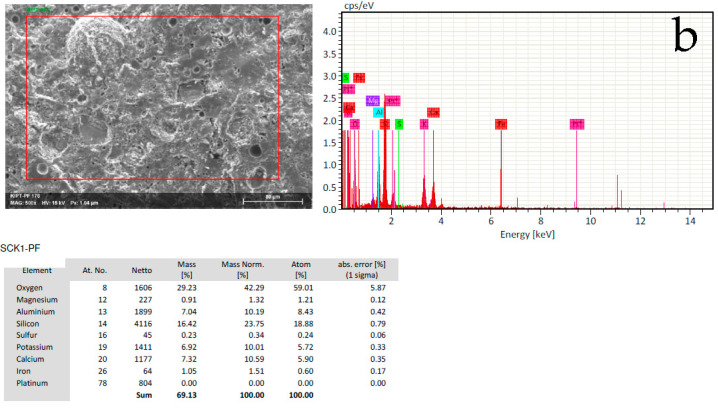
SEM-EDS analysis of BFS samples without RLOW simulants: (**a**) pre- and (**b**) post-fire tests.

**Figure 9:**
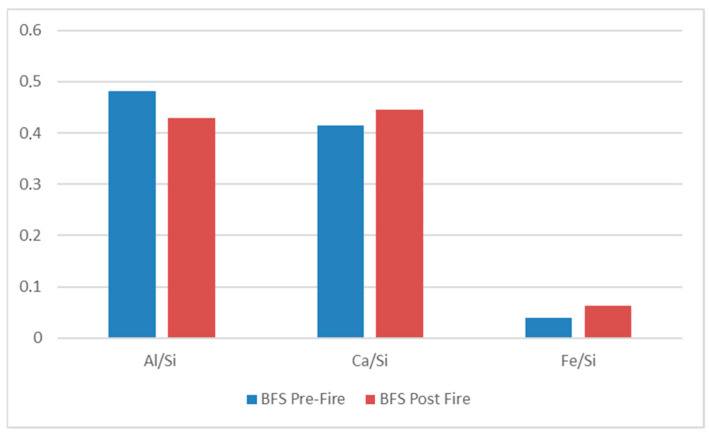
Comparison of mass % composition of the elements between pre- and post-fire BFS samples obtained by SEM-EDS analyses.

Figure 8 and Figure 9 summarize the EDS results of pre-and post-fire BFS samples without oil addition.

The elemental ratios calculated from EDS data before and after fire exposure—namely Al/Si, Ca/Si, and Fe/Si show only modest variations (all within ±0.05 on a mass ratio basis). Given the semi-quantitative nature of EDS and its sensitivity to sampling area, these differences are not considered significant. It is concluded that there was no significant elemental redistribution in the matrix after thermal exposure. This suggests that the samples retained their overall structural integrity [3,24].

The results demonstrate that thermal exposure leads to significant elemental redistribution, consistent with the observed shrinkage and cracking in fire-exposed samples. These changes are attributed to dehydration, gel densification, and loss of volatile components. This analysis supports the importance of chemical stability and thermal resistance in geopolymer formulation to mitigate shrinkage-related failures under elevated temperatures.

## 4. Conclusions

This study investigated the thermal and mechanical behavior of three geopolymer formulations, metakaolin-based (MK), blast furnace slag-based (BFS), and a MK-BFS-fly ash ternary blend (MIX), used for direct incorporation of surrogate RLOWs. The selected matrices were subjected to accelerated ageing through climatic chamber cycles and fire resistance testing to simulate potential environmental and accidental scenarios relevant to nuclear waste immobilization.

UNIPI’s characterizations demonstrated that BFS-based and MIX matrices provide superior mechanical, thermal, and environmental resilience compared to MK-based geopolymers, especially when incorporating RLOW surrogates.

Initial results on the thermal behavior of the formulations tested (MK and MIX) show the microcracking of the matrices under thermal cycling.

The pronounced sensitivity of MK-based formulations to both organic inclusion and fire/elevated temperature highlights the importance of binder selection for RLOW conditioning: the matrix’s massive cracking may lead to higher waste release.

The Al/Si, Ca/Si, and Fe/Si ratios obtained by EDS before and after fire exposure differed by no more than ±0.05 (mass ratio), which falls inside the typical semi-quantitative error and sampling variability of the method. Hence, no statistically significant elemental redistribution occurred, indicating the matrix preserved its overall structural integrity after thermal exposure.

Nevertheless, the quantity of available data and post-mortem analysis remains insufficient to draw a robust conclusion.

Behavior at high temperatures and under fire-like conditions needs to be studied in greater depth due to the important role of the thermal gradients in reducing the strength capacity of these matrices. Further leaching tests will also be taken into consideration to evaluate the immobilization performance and leach resistance. A full follow-up campaign is already scheduled within the EURAD-2 project to complete the durability matrix required for regulatory qualification.

## Figures and Tables

**Table 1:** RLOW types in EU member states [5].

Organisation	Country	Lubricants	Organic Solvents	Scintillation Cocktail	Decontamination Liquids	Sludge
KIPT	Ukraine	Y				
Sellafield Ltd.	UK		Y			
CV Rez	Czech Republic	Y	Y	Y	Y	
ÚJV Rez	Czech Republic	Y	Y	Y		
SOGIN	Italy	Y	Y			
RATEN	Romania	Y		Y		
Cernavoda NPP	Romania	Y	Y	Y		Y

**Table 2:** Composition (in wt%) of the different geopolymer formulations investigated and RLOW type and loading amount in each matrix (in vol%).

Sample ID	MK (wt%)	BFS (wt%)	FA (wt%)	Quartz Sand (wt%)	KOH (wt%)	K_2_SiO_3_ (wt%)	NaOH (wt%)	Na_2_SiO_3_ (wt%)	H_2_O (wt%)	RLOW (vol%)
MK	32.5	-	-	-	10	40.5	-	-	17.0	-
MK-N	32.5	-	-	-	10	40.5	-	-	17.0	20.0 (Nevastane)
MK-T	32.5	-	-	-	10	40.5	-	-	17.0	20.0 (TBP/dodecane)
BFS	-	46.5	-	28.0	-	-	5.5	1.5	18.5	-
BFS-T	-	46.5	-	28.0	-	-	5.5	1.5	18.5	20.0 * (TBP/dodecane)
MIX	14.0	20.0	34.0	-	9.0	11.0	-	-	12.0	-
MIX-S	14.0	20.0	34.0	-	9.0	11.0	-	-	12.0	20.0 ** (Shell Spirax)

* Tween 80 surfactant used: 0.5 vol% relative to the waste volume. ** Castament FW10 additive used to improve several properties (e.g., homogeneity, compressive strength, etc.): 0.5 vol% relative to the waste volume.

**Table 3:** Crush load, ultimate stress, and the relative standard deviations of geopolymer samples tested under compression.

Sample ID	Crush Load (kN)	Crush Load Standard Deviation	Ultimate Stress (MPa)	Ultimate Stress Standard Deviation
MK	22.33	7.48	11.38	3.81
MK-N	17.91	1.85	9.13	0.95
MK-T	21.04	5.85	10.72	2.98
BFS	24.43	4.33	12.45	2.21
BFS-T	- *	- *	- *	- *
MIX	23.32	3.56	11.88	1.82
MIX-S	11.05	1.05	5.63	0.54

* Data not available.

**Table 4:** Crush load, ultimate stress, and strength loss of MK-based and MIX-based samples after thermal ageing in climatic chamber tested under compression.

Sample ID	Crush Load (kN)	Crush Load Standard Deviation	Ultimate Stress (MPa)	Ultimate Stress Standard Deviation	Strength Loss (%)
MK	17.69	3.08	9.01	1.57	20.76
MK-N	12.96	1.45	6.61	0.74	27.63
MK-T	7.79	0.50	3.97	0.30	63.00
MIX	26.27	6.76	13.39	3.45	−12.68
MIX-S	13.10	0.17	6.68	0.09	−18.51

**Table 5:** Ultimate load for fresh and AFL samples and strength loss of MK-based and MIX-based samples.

Sample ID	Ultimate Stress Fresh (MPa)	Ultimate Stress AFL (MPa)	Strength Loss (%)
MK	15.36	0	100
	8.38	0	100
BFS	10.48	3.15	69.95
	10.23	2.00	80.47
MIX	10.24	6.02	41.19
	14.65	4.71	67.87

## Data Availability

The original contributions presented in this study are included in the article/Supplementary Materials. Further inquiries can be directed to the corresponding author.

## Supplementary Materials

The following supporting information can be downloaded at: https://www.mdpi.com/article/10.3390/ma18133181/s1, Figure S1: Compression test execution: (a) initial loading phase and (b) brittle rupture [18]; Table S1: Chemical composition (wt%) of raw materials used for geopolymers formulation determined from energy dispersive X-ray fluorescence (XRF) spectrometry by PREDIS project partners [2]; Figure S2: Thermal ageing set up [18].
