# Supplementary material for: Durability Assessment of Alkali-Activated Geopolymers Matrices for Organic Liquid Waste Immobilization
Source: Materials (Basel). 2025 Jul 4;18(13):3181. doi: 10.3390/ma18133181 (PMC12251448; doi:10.3390/ma18133181)
Supplement: Supplementary file 1 [file materials-18-03181-s001.zip › materials-3662188-supplementary.pdf]

Supplementary Materials

# DDurability Assessment of Alkali-Activated Geopolymers Matrices for Organic Liquid Waste Immobilization

**Rosa Lo Frano [1,*], Salvatore Angelo Cancemi [1], Eleonora Stefanelli [1] and Viktor Dolin [2]**

[1] Dipartimento di Ingegneria Civile e Industriale, Università di Pisa, Largo Lucio Lazzarino 2, 56122 Pisa, Italy; salvatore.cancemi@unipi.it (S.A.C.); eleonora.stefanelli@unipi.it (E.S.)
[2] State Institution "Institute of Environmental Geochemistry of National Academy of Sciences of Ukraine", 34a, Palladin Av., 03680 Kyiv, Ukraine; vdolin@ukr.net
* Correspondence: rosa.lo.frano@unipi.it

**Table S1.** Chemical composition (wt%) of raw materials used for geopolymers formulation determined from energy dispersive X-ray fluorescence (XRF) spectrometry by PREDIS project partners [2].

| **Material** | **$SiO_2$** | **$Al_2O_3$** | **CaO** | **MgO** | **$SO_3$** | **$Na_2O$** | **$K_2O$** | **$TiO_2$** | **$P_2O_5$** | **MnO** | **$Fe_2O_3$** |
|---|---|---|---|---|---|---|---|---|---|---|---|
| MK (Metamax®, BASF, UK) | 54.1 | 42.5 | 0.1 | < 0.1 | 0.2 | 0.1 | 0.2 | 2 | 0.1 | < 0.1 | 0.5 |
| BFS (Ecocem Benelux, BE) | 33.6 | 8.7 | 49.0 | 5.1 | 1.5 | - | 0.4 | 0.8 | - | 0.3 | 0.4 |
| MK (Ukrainian) | 51.0 | 35.5 | 0.25 | 0.16 | - | 0.15 | 0.27 | 0.78 | - | - | 1.05 |
| BFS (Ukrainian) | 40.6 | 6.02 | 45.1 | 3.61 | 1.74 | 0.42 | 0.40 | 0.30 | 0.01 | 0.17 | 0.62 |
| FA (Ukrainian) | 46.1 | 18.0 | 4.10 | 1.46 | 0.21 | 2.10 | | 1.78 | - | 0.14 | 22.1 |
| Quartz sand | 98.50 | 0.50 | 0.10 | 0.02 | - | 0.05 | 0.05 | 0.08 | - | - | 0.05 |

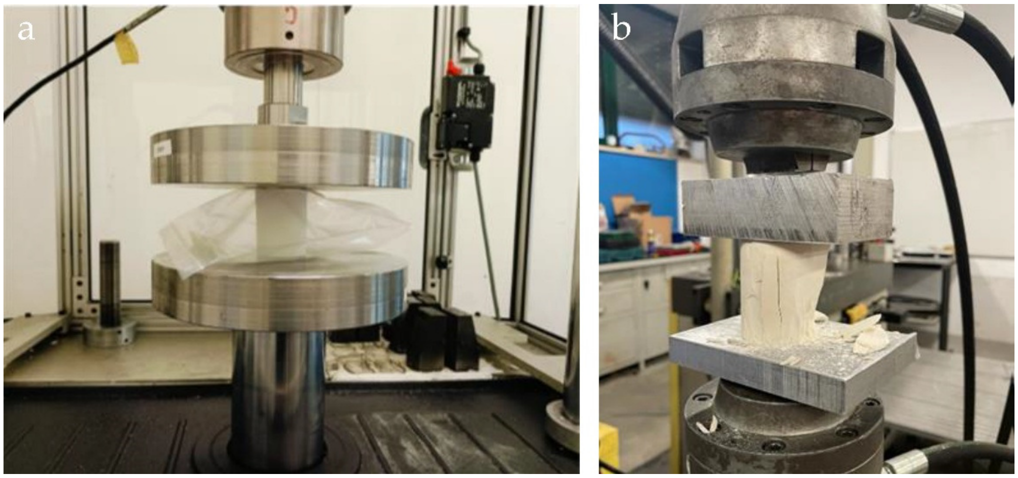


**Figure S1.** Compression test execution: (a) initial loading phase and (b) brittle rupture [18]

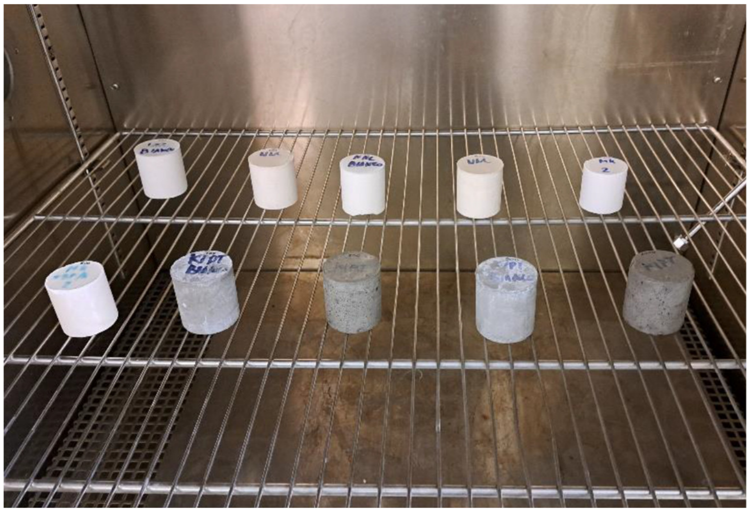

**Figure S2.** Thermal ageing set up [18]
